# Membrane protein-chimeric liposome-mediated delivery of triptolide for targeted hepatocellular carcinoma therapy

**DOI:** 10.1080/10717544.2021.1983072

**Published:** 2021-09-27

**Authors:** Yanwen Zheng, Fanhua Kong, Songyang Liu, Xi Liu, Dongni Pei, Xiongying Miao

**Affiliations:** a Department of Liver Surgery, The Second Xiangya Hospital of Central South University, Changsha, China; b Institute of Hepatobiliary Diseases of Wuhan University, Transplant Centre of Wuhan University, Zhongnan Hospital of Wuhan University, Wuhan University, Wuhan, China; c College of Biology, State Key Laboratory of Chemo/Biosensing and Chemometrics, Key Laboratory for Bio-Nanotechnology and Molecular Engineering of Hunan Province, Hunan University, Changsha, China; d Department of Gastrointestinal Surgery, The Third Xiangya Hospital of Central South University, Changsha, China

**Keywords:** Triptolide, hepatocellular carcinoma, drug delivery, membrane proteins, apoptosis, necroptosis

## Abstract

Triptolide (TPL) is a diterpenoid triepoxide with broad antitumor efficacy, while lack of mechanism of action, severe systemic toxicity, and poor water solubility of TPL limited its usage. To unveil the mechanism of action and improve the pharmaceutical properties of TPL, here we explored the molecular mechanism of TPL and then fabricated TPL-loaded membrane protein-chimeric liposomes (TPL@MP-LP) and tested its anticancer efficacy against hepatocellular carcinoma (HCC). CCK8 assay, colony formation assay, EdU assay, and flow cytometry were used to examine the activity of TPL. RNA sequence and gain-and-loss of function assays were used to explore the molecular mechanisms. TPL@MP-LP was characterized by size, zeta potential, polydispersity index, and transmission electron microscopy. Cellular uptake and cell viability assay were performed to evaluate the internalization and anticancer efficacy of TPL@MP-LP *in vitro*. Biodistribution and *in vivo* antitumor efficacy of TPL@MP-LP were evaluated on orthotopic HCC mice models. TPL robustly inhibited HCC cells by inducing cell proliferation arrest, apoptosis via the mitochondrial pathway, and necroptosis via RIPK1/RIPK3/MLKL signaling. TPL was successfully loaded into MP-LP, with a drug-loading capacity of 5.62 ± 0.80%. MP-LP facilitated TPL internalization and TPL@MP-LP exerted enhanced anticancer efficacy against Huh7 cells. TPL@MP-LP showed targeting ability to the tumor site. More importantly, TPL@MP-LP treatment suppressed tumor growth but showed minimal damage to liver and renal functions. TPL exerted anticancer effects on HCC via inducing cell proliferation arrest, apoptosis, and necroptosis, and the MP-LP might be a promising delivery strategy to improve the antitumor efficacy while mitigating toxicity of TPL for HCC therapy.

## Introduction

Hepatocellular carcinoma (HCC) is the most common form of primary liver cancer and a main cause of cancer-associated death worldwide (Ferlay et al., 2019; Siegel et al., 2021). HCC usually develops from viral hepatitis, cirrhosis, or nonalcoholic fatty liver disease (Daher et al., 2018). Surgical resection is preferable for HCC treatment; however, clinical management of HCC has been challenging as most patients are diagnosed at advanced stages and classified as not suitable for surgery (Forner et al., 2018). Conventional chemotherapy has been the mainstay for HCC treatment, but severe systemic toxicity limited the application of most chemotherapeutics and the prognosis of chemotherapy of HCC is unsatisfactory (Anwanwan et al., 2020). The exploration of new drugs and the development of nanomedicine offered a great number of alternatives to increase treatment efficacy and enhance drug delivery for targeted therapy of HCC (Elnaggar et al., 2021; Wu et al., 2021). Therefore, it is of great clinical significance to find a novel therapeutic agent and develop an efficient therapeutic strategy for treating HCC, overcoming the limitations of chemotherapy.

Triptolide (TPL) is a diterpenoid triepoxide purified from the Chinese herb Tripterygium wilfordii Hook F (Kupchan et al., 1972). TPL has been found to be highly effective against various malignant tumors such as pancreatic cancer (Phillips et al., 2007), breast cancer (Li et al., May 2015) and melanoma (Jao et al., 2016) via various mechanisms (Hou et al., 2019; Wang et al., 2020). TPL can induce apoptosis, upregulate tumor suppressors and downregulate carcinogens, interact with RNA polymerase II complex, and modulate tumor microenvironment (Hou et al., 2018). While the underlying molecular mechanisms of TPL on HCC were still elusive, their potential therapeutic efficacy is worthy of further exploration. Furthermore, TPL is extremely toxic with very poor water solubility and low bioavailability, which limited its clinical application (Xu et al., 2013; Noel et al., 2019). Over the past decades, nanomedicine-based strategies have been emerging for improving the bioavailability of TPL while reducing adverse toxicity (Ren et al., 2021). Natural nanovesicles such as exosomes are emerging delivery systems for the efficient delivery of chemicals, nucleic acids and proteins (Yurkin & Wang, 2017; Shao et al., 2020), and may be used for TPL delivery (Jiang et al., 2021). However, the development of exosomes for drug delivery has been challenging for the lack of standard isolation and purification protocols and large-scale production (Li et al., 2019). Besides, efficient cargo loading into exosomes is not feasible (Lener et al., 2015; Kooijmans et al., 2017). Therefore, the modification of current delivery vesicles to mimic exosomes may provide strategies for addressing the limitations of exosomes (Leggio et al., 2020).

Liposomes are widely used nano vectors for drug delivery. Liposomal nanoformulations are the most successful nanomedicines family member (Crommelin et al., 2020). Hydrophobic substances such as TPL can be encapsulated in liposomes with improved bioavailability. Nanoliposomes have phospholipid bilayer and 100-nm nanosize, which are also features of exosomes. Interestingly, it has been reported that transmembrane domains of cell membrane proteins could be used for surface modification of nanoliposomes (Zhang et al., 2019) to improve circulation and enhance tumor-targeting properties. Hence, membrane proteins chimeric nanoliposomes are similar to exosomes structurally and may be used for advanced drug delivery. Here, we prepared TPL-loaded membrane proteins chimeric liposomes (TPL@MP-LP) for enhancing antitumor efficacy and reducing the toxicity of TPL. TPL exerted anticancer effects on HCC via inducing cell proliferation arrest, apoptosis, and necroptosis. TPL@MP-LP exhibited increased therapeutic efficacy against HCC in mice with prolonged survival but minimal damage to normal tissues.

## Methods

### Cell culture

Human hepatoma cell lines Huh7, Huh7-luc, and Hep3B cells were obtained from ZSBIO (Beijing, China), which also performs cell line STR genotyping. Huh7-luc cells were purchased from BnBio (Beijing, China). Both cells were cultured with DMEM supplemented with 10% FBS and 1% penicillin-streptomycin in a humidified incubator with 5% CO_2_ at 37 °C.

### Cell viability analysis

Cell viability was determined by CCK8 kit (GeneView, China). 1 × 10^4^ cells were seeded into 96-well plates and cultured for 24 h, followed by adding different concentrations of TPL (Yuanye, Shanghai, China) for incubation for 24 h. A microplate spectrophotometer (Thermo Fisher, USA) was used to determine the OD values at 450 nm.

### Colony formation assay

About 5000 cells were seeded onto a six-well plate for incubation of 4 days. Then TPL was added as indicated for another incubation of 3 days. The colony was washed by PBS (phosphate buffered saline) two times, fixed with 4% paraformaldehyde, and stained with 1% crystal violet staining solution (Beyotime, China). Images of colonies were captured by a digital camera.

### Western blot

Cell samples were lysed in RIPA buffer supplemented by protease and phosphatase inhibitors (TargetMol, USA). The proteins were denatured by heating, added to the chamber for electrophoresis, and electrotransfered onto the PDVD membrane. After blocking with 3% bovine serum albumin for 1 h, the membrane was incubated with primary antibody overnight at 4 °C. After washing with TBST three times, second antibody was added and incubated for 1 h at room temperature. The signal was detected by an enhanced chemiluminescence system (Life Tec, USA). The primary antibodies were listed as following: caspase 3/cleaved caspase 3 (1:1000; CST, USA), caspase 9/cleaved caspase 9 (1:1000; CST, USA), Bad (1:1000; Abclonal, China), p-MLKL (1:1000; Abclonal, China), MLKL (1:1000; Abclonal, China), p-RIPK1 (1:1000; Abclonal, China), RIPK1 (1:1000; Abclonal, China), RIPK3 (1:1000; Abclonal, China), p-RIPK3 (1:1000; Abclonal, China), BIRC3 (1:1000; Abclonal, China), CYLD (1:1000; Abclonal, China), and GAPDH (1:1000; Abclonal, China).

### EdU assay

EdU assay was performed using an EdU kit (Beyotime, China). HCC cells were seeded in 24-well plates for incubation for 2 days. Then, the cells were rinsed once with PBS, incubated with EdU mix for 2 h at 37 °C. After permeabilization with 0.25% Triton X-100 (GeneView, China), the cells were incubated with Click Reaction Solution for 30 min, stained with DAPI, and captured using a fluorescence microscope (Olympus Inc., USA).

### Flow cytometry assay

Apoptotic rates of HCC cells treated with TPL were determined by flow cytometry using a kit purchased from Beyotime (China) and performed according to the manufacturer’s institution.

### RNA-sequencing (RNA-seq)

RNA-seq was performed by Novogene (Beijing, China). Briefly, RNA was extracted, the cDNA libraries were generated using the NEBNext® UltraTM RNA Library Prep Kit, then the cDNA was sequenced by an Illumina Novaseq platform and 150 bp paired-end reads were generated. After quality control of data, the sequences reads were paired with the Reference genome, and the expression profiles were performed by featureCounts.

### Preparation of TPL@MP-LP

Cell membrane proteins from Huh7 liver cancer cells were extracted using a Mem-PER™ Plus Membrane Protein Extraction Kit (ThermoFisher, USA). The amount of membrane protein extracted was determined by the BCA assay (Beyotime, China).

Liposomes were constructed using the thin layer evaporation method. A total of 15 mg of 1,2-Dimyristoyl-sn-glycero-3-phosphocholine (DMPC), cholesterol, and 1,2-distearoyl-sn-glycero-3-phosphoethanolamine-N-[methoxy(polyethylene glycol)-2000] (DSPE-PEG2000) at the mass ratio of 10:4:1 were dissolved in 4 mL of chloroform and methanol (3:1, v/v). Solvents were evaporated under reduced pressure to form a thin film, followed by hydrating with water for 15 min. Lipids in water were extruded through 200-nm and 100-nm membranes (Whatman) using a Mini Extruder (Avanti). To construct membrane protein-chimeric liposomes (MP-LP), cell membrane proteins (80 ug) were added to the lipid suspension during hydration, followed by the extruding procedures. Triptolide-loaded MP-LP (TPL@MP-LP) was constructed by adding 1 mg of triptolide to the lipids before thin layer formation. Excessive free TPL was removed by dialysis (30 kDa, 1:400, v/v) for an hour.

### Characterization, drug loading, and release

Sizes, zeta potentials, and polydispersity index (PDI) of liposomes and MP-LP were measured by Zetasizer Nano (ZS90, Malvern, UK). Morphology of liposomes and MP-LP were observed using a Tecnai G2 Spirit transmission microscope (FEI) after staining with 2% (w/v) phosphotungstic acid. Coomassie blue staining (Beyotime, China) was performed to investigate the protein profiles of MP-LP. To evaluate the stability of liposomes and MP-LP, size, and PDIs were measured after storage, liposomes were stored at 4 °C while MP-LP were stored at −80 °C after vacuum freezing and drying.

The amount of TPL encapsulated in MP-LP was measured using high-performance liquid chromatography (HPLC) system (H20AT SHIMADZU, Japan) equipped with an Agilent TC-C18 column (250 mm ×4.6 mm, 5 μm, Agilent). A mobile phase of acetonitrile: water = 40:60 at a flow rate of 1.00 mL/min at column temperature of 30 °C was used. Absorbance at 218 nm was detected to measure the concentration of TPL.

To measure TPL release, 50 mg of TPL@MP-LP in 1 mL PBS was placed in a dialysis bag (30 kDa). The bag was immersed in a beaker containing 25 mL of PBS at 37 °C with stirring (200 rpm). Samples (500 μL) were collected at 1, 2, 4, 6, 8, 12, 24, 36, and 48 h post placing and were analyzed by HPLC.

### Cellular uptake

For cellular uptake studies, liposomes and MP-LP were labeled with Dio (green, Yeason, China) similar to DiR labeling. Huh7 cells were treated with Dio-liposomes or Dio-MP-LP for 0.5, 1, 2, and 4 h followed by paraformaldehyde-fixing and nuclei staining (DAPI, Beyotime, China). Cellular internalization of Dio-liposomes or Dio-MP-LP in Huh7 cells was observed by an IX73 fluorescence microscope (Olympus, Japan). The fluorescence intensity of Dio-liposomes and Dio-MP-LP were analyzed and compared.

### Biodistribution and tumor-targeting

To observe biodistribution of MP-LP *in vivo*, DiR-labeled MP-LP was developed by adding 10 μg of DiR dye (Yeason, China) to dissolved lipids (10 mg) before the formation of thin layer. Orthotopic HCC animal model was developed by injecting 1 × 10^6^ Huh7-luc cells in 20 μL PBS with matrigel (1:1, BD, USA) into the liver of nude mice. Mice with HCC were given 100 μL of free DiR, LP-DiR or MP-LP-DiR (5 mg/kg) via i.v. injection. *In vivo* and *ex vivo* biofluorescence images were obtained using IVIS spectrum (PerkinElmer, USA) 8 and 24 h post-administration.

### Antitumor efficacy *in vivo*

Animal studies were approved by the ethical committee of the Department of Laboratory Animal of Central South University. Animals were housed following IACUC guidelines. As the incidence of HCC in males is higher than in females, we generated orthotopic HCC mice models in male BALB/C nude mice (6 weeks, Hunan Slack Scene of Laboratory Animal Co., Ltd.). Seven days after xenografts generation, mice were randomly divided into four groups: PBS, TPL, TPL@LP, and TPL@MP-LP group.

All formulations were i.v. injected four times with an interval of three days at an equivalent dose of TPL (100 μg/kg). Bioluminescence showing the size of liver tumor was obtained by IVIS. Bodyweight was measured every 2 days. After the treatment, mice were sacrificed and organs and blood samples were collected. Major organs and tumors were subjected to histopathological assessment, serum levels of ALT, AST, Cr, and BUN were measured using assay kits (Huili Biotech, China) according to the manufacturer’s instructions.

### Immunohistochemical (IHC) staining

The IHC analyses of animal samples were performed as previously described. 4-μM-thick slices were prepared from paraffin-embedded tissues, incubated with 3% H_2_O_2_, and then ready for epitope retrieval. The slices were incubated with cleaved caspase 3 and Ki67 (CST, USA) antibodies overnight. On the following day, the slices were washed by PBS, incubated with second antibody, stained by DAB, and counterstained by hematoxylin. Finally, the slices were dehydrated and mounted with neutral balsam.

### Statistical analysis

Data were presented as mean ± SD. Student's *t*-test was performed for comparison between two groups. A one-way analysis of variance (ANOVA) test was performed for comparison among three or more groups. Statistical significance level α was set at 0.05.

## Results and discussion

### TPL inhibited HCC cells via inducing cell proliferation arrest, apoptosis, and necroptosis

**Figure 1.**
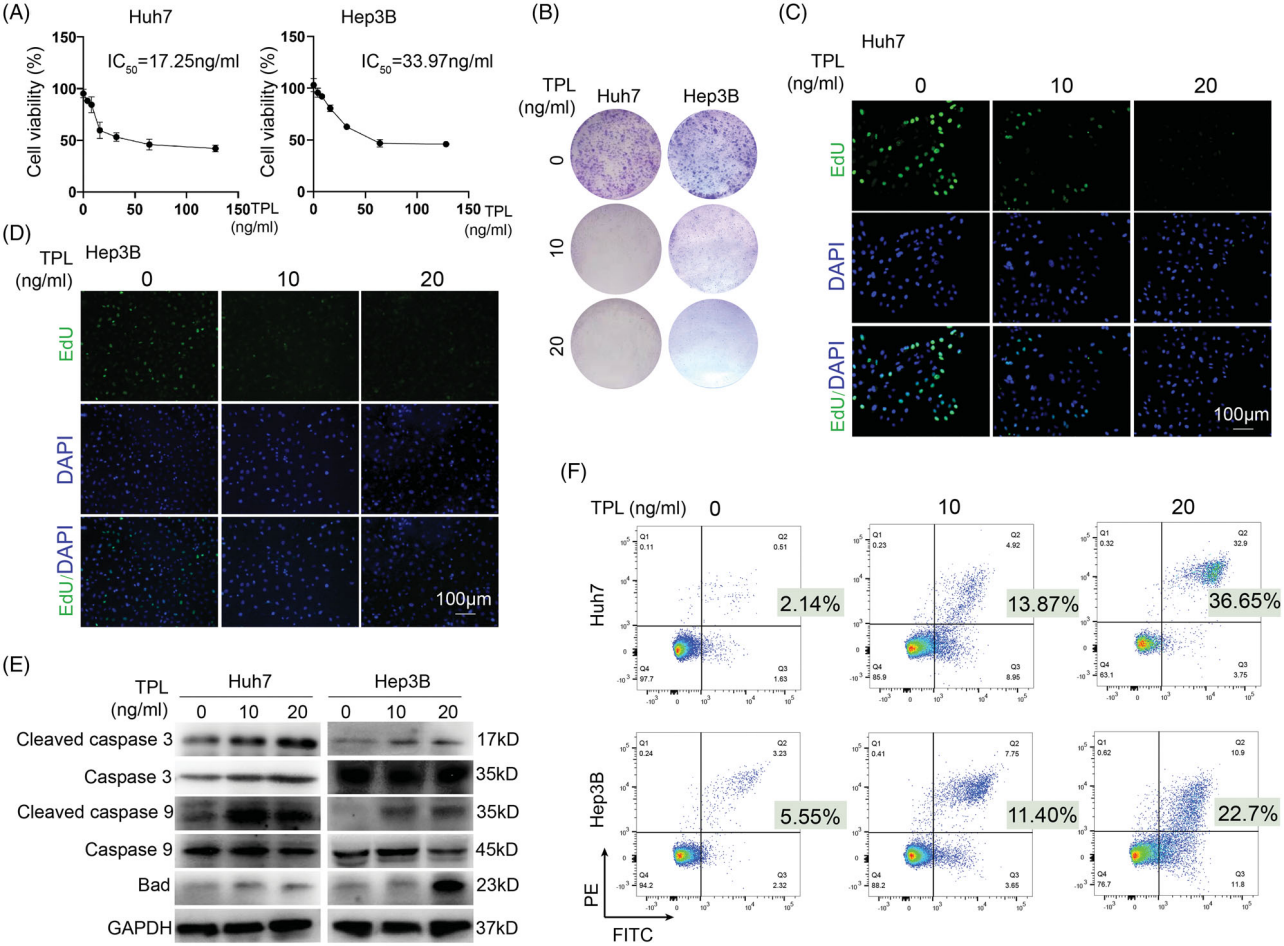
*In vitro* cytotoxicity of TPL on HCC. (A) CCK-8 was used to determine the killing effect of TPL on Huh7 and Hep3B cells. (B) TPL (10 and 20 ng/ml) markedly inhibited colony formation of Huh7 and Hep3B cells. (C, D) TPL markedly inhibited cell proliferation of these two cells. (E) Apoptosis-associated proteins, including cleaved caspase 3/9 and Bad were induced by TPL in a dose-dependent pathway. (F) Flow cytometry showed markedly induced apoptosis by TPL in Huh7 and Hep3B cells. The bar indicated 100 μm.

To determine the kill effect of TPL in liver cancer cells, cell viability of Huh7 and Hep3B cells was detected by CCK8 assay in various concentrations of it. The half-maximal inhibitory concentration (IC_50_) of TPL was 17.25 and 33.97 ng/ml in Huh7 and Hep3B cells, respectively (Figure 1(A)). Colony formation assay showed a remarked inhibition of TPL on colony formation in both cells (Figure 1(B)). EdU assay further showed an obvious inhibition of cell proliferation in both cells (Figure 1(C,D)). Then, we detected a set of apoptosis-associated proteins in liver cancer cells after treatment of TPL. Cleaved caspase 3, cleaved caspase 9, and Bad were consistently upregulated by TPL in a concentration-based pathway in Huh7 and Hep3B cells (Figure 1(E)), suggesting that TPL induced apoptosis through the mitochondrial pathway. Quantitatively, cell apoptosis rate was determined by flow cytometry and TPL (10 ng/ml) led to 13.87 and 11.4% apoptotic cell death in Huh7 and Hep3B cells, respectively, and further increased to 36.65 and 22.7% by 20 ng/ml TPL (Figure 1(F)).

**Figure 2.**
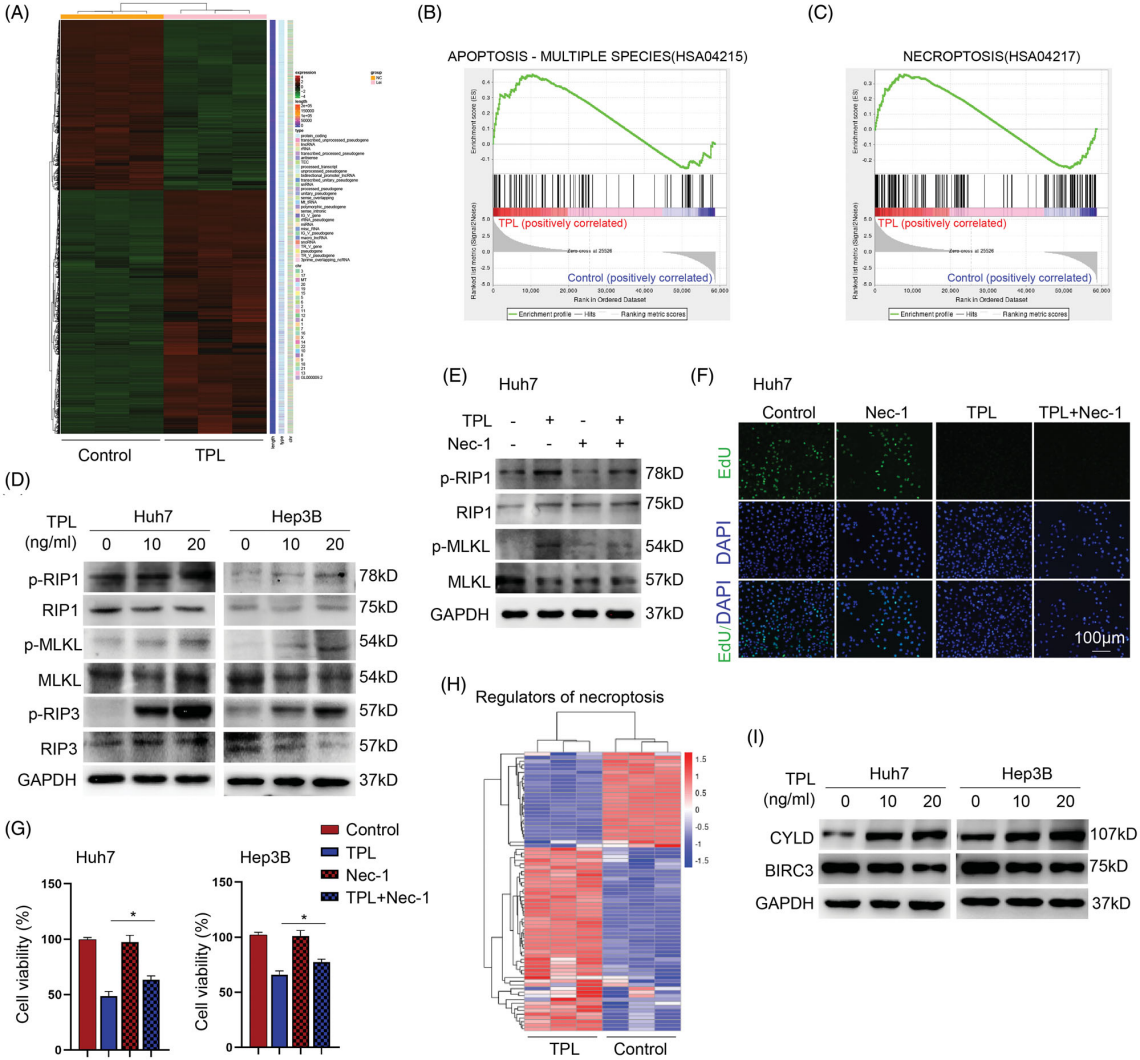
Antitumor mechanisms of TPL. (A) Heat map showed the transcriptome profiles of Huh7 cells treated with TPL (20 ng/ml) or not. (B, C) GSEA analysis showed that TPL led to the enrichment of apoptosis and necroptosis in Huh7 cells. (D) The key regulators of necroptosis, p-RIP1, p-RIP3 and p-MLKL were upregulated by TPL in a dose-dependent pathway in Huh7 and Hep3B cells. (E) Nec-1 (40 μM) inhibited the activity of p-RIP1 and p-MLKL induced by TPL. (F) Pretreatment of nec-1 did not improve the cell proliferation of Huh 7 cells in response to TPL (20 ng/ml). (G) Pretreatment of nec-1 improved the cell viability of Huh7 cells in response to TPL (20 ng/ml). (H) The Heat-map showed the differentiated necroptosis genes (belonging to the HSA04217 gene set) induced by TPL in Huh7 cells. (I) CYLD was upregulated by TPL while BIRC3 was downregulated by it in Huh7 and Hep3B cells. The bar indicated 100 μm. Nec-1, necrostatin-1; *p < .05.

To explore the underlying mechanisms of TPL on liver cancer cells, we analyzed global gene expression variations by RNA-seq in Huh7 cells after treatment of TPL (Figure 2(A)). Expectedly, Gene Set Enrichment Analysis (GSEA) showed that TPL led to significant enrichment of apoptosis pathway (Figure 2(B)). Furthermore, we also observed a significant enrichment of necroptosis gene set exerted by TPL in Huh7 cells (Figure 2(C)). Necroptosis is regulated by RIPK1, RIPK3, and MLKL. We observed that TPL increased the phosphorylation of these proteins by a concentration relied pathway in Huh7 and Hep3B cells (Figure 2(D)). Necrostatin-1 (Nec-1) is a specific inhibitor of necroptosis by blocking RIPK1 activation. Administration of Nec-1 markedly inhibited the activation of RIPK1 and the downstream MLKL in both cells (Figure 2(E)). Nec-1 could not rescue the inhibition of cell proliferation by TPL (Figure 2(F)), while increased cell viability was inhibited by TPL in Huh7 cells (Figure 2(G)). Furthermore, RNA-seq analysis unveiled that several key regulators of necroptosis were dysregulated upon TPL (Figure 2(H)). Among them, CYLD which is responsible for the deubiquitination of RIPK1 was significantly upregulated, and BIRC3 playing a reverse role on RIPK1 was downregulated. CYLD was upregulated by TPL while BIRC3 was downregulated and BIRC3 playing a reverse role on RIPK1 was downregulated (Figure 2(I)). Thus, these data indicated that TPL induced apoptosis and necroptosis in liver cancer cells via the mitochondrial pathway and RIPK1/RIPK3/MLKL signaling.

TPL is noted for its multiple pharmacological effects, such as anti-inflammatory, anti-cancer, and immunoregulation. TPL exhibits promising anticancer effects in acute myeloid leukemia and various solid cancers. A large number of molecular targets and signaling pathways were correlated with the effect of TPL (Li et al., 2014). TPL could lead to cell growth arrest and mediate cell death by inducing apoptosis and autophagy (Krosch et al., 2013). For instance, TPL inhibited the expression of HSP70, a chaperone, via miR-142-3p and induced apoptosis in gastric and pancreatic cancer cells (MacKenzie et al., 2013). Our study unveiled that TPL induced apoptosis through a mitochondrial pathway in HCC cells. Furthermore, we also demonstrated an essential role of necroptosis playing in TPL-mediated cell death via RNA-seq and gain-and-loss of function assays in HCC. Necroptosis is reported to be an alternative mechanism to restrict tumor development, and the key regulators might drive an imbalance of the immune system or endothelial cells to promote metastasis (Su et al., 2015). It should be noted that Huh7 and Hep3B cells responded differently to TPL. We speculate that the effect of TPL was influenced by the expression level of IDH1. It has been reported that TPL disrupts glutathione *de novo* synthesis, and establishes synthetic lethality with intrinsic oxidative stress in IDH1-mutated cells, which leads to oxidation of macromolecules, such as DNA oxidative damage and lipid peroxidation (Yu et al., 2020). High expression of IDH1 may be an indication of poor response to TPL in cancer cells. We retrieved the expression profile of GSE151412 and found that IDH1 of Huh7 cells was significantly higher than that of Hep3B (Supplementary Figure S1A).

We also demonstrated an essential role of necroptosis in TPL-mediated cell death by RNA-seq and gain-and-loss of function assays in HCC. Necroptosis is reported be to an alternative mechanism to restrict tumor development, and the key regulator of necroptosis may induce an imbalance of immune system or promote metastasis. Necroptosis induced by TPL is regulated by CYLD and BIRC3. The expression of CYLD can be induced by various factors, such as TNF-α, IL-1β, and lipopolysaccharide (Sun, 2010). It has been demonstrated that RANKL, BAF57, and SNAIL can regulate CYLD expression by activating condition-dependent pathway (Jono et al., 2004; Lim et al., 2007; Sun, 2010). In TPL-induced necroptosis of Huh7 and Hep3B cells, we found a nearly 10-fold increased expression of RANKL mRNA compared to control but not for BAF57 and SNAIL (Supplementary Figure S1B). Previous reports showed that RANKL is essential for cancer development by distinct mechanisms (Rao et al., 2018). Thus, we speculated that RANKL might be an activator of CYLD upon TPL treatment in HCC cells, but further exploration is needed to demonstrate the link. On the other hand, previous works demonstrated that the transcriptional factor, NFKB1 was responsible for the induction of BIRC3 in several tumor cells (Cartwright et al., 2016). Our transcriptome data and PCR data showed a significant decrease of NFKB1 upon TPL treatment (Supplementary Figure S1C), inducating that NFKB1 might be the target of TPL for inhibition of BIRC3 in HCC cells. However, it should be noted that TPL is not a well-assembled drug for the treatment of cancer, due to the poor water solubility, low bioavailability, and large side effects.

### Characterization of nanoparticles

**Figure 3.**
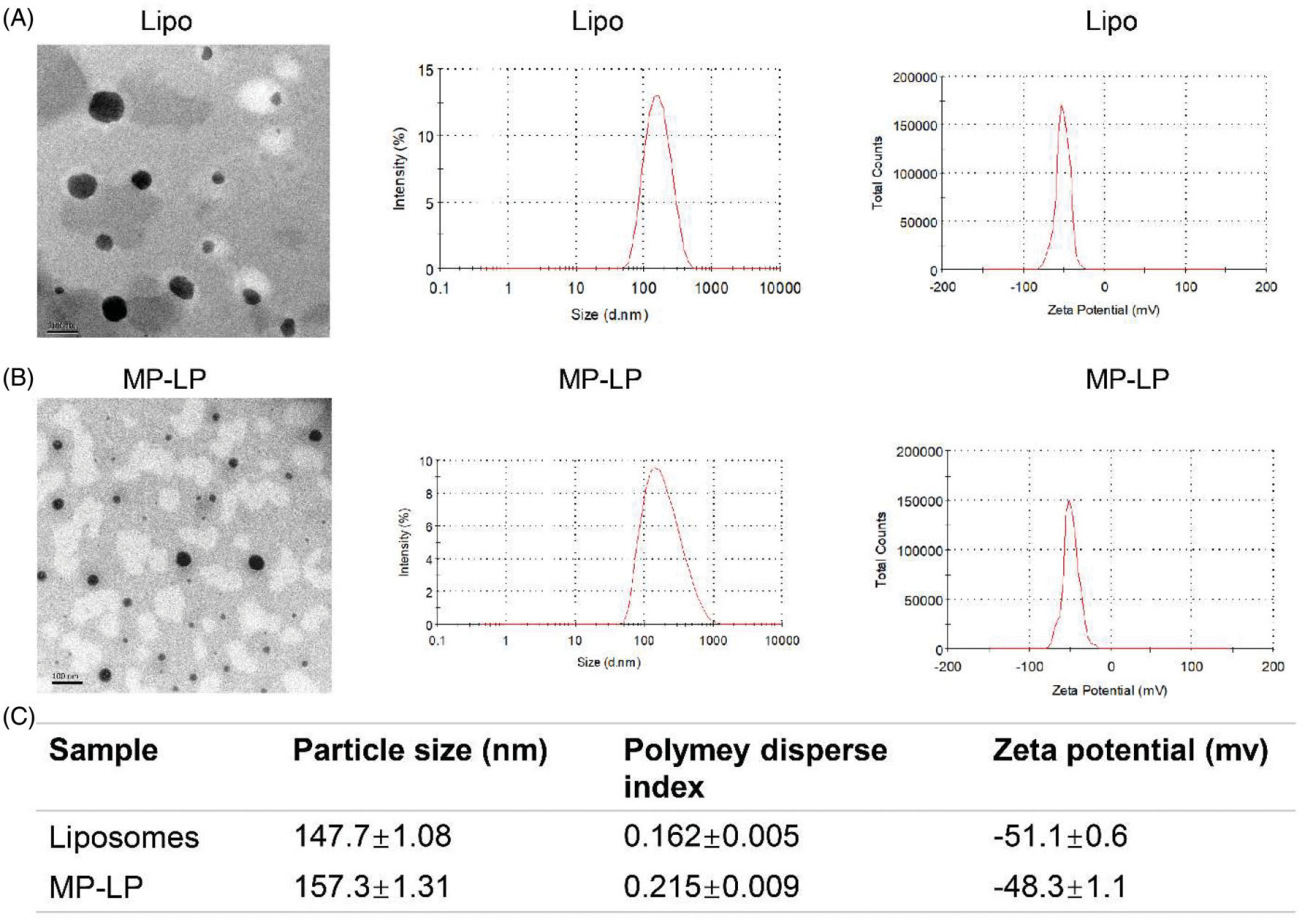
Characterization of liposomes and MP-LP. (A) TEM images, size distribution by dynamic light scattering and zeta potential of Lipo. (B) TEM images, size distribution by dynamic light scattering and zeta potential of MP-LP. (C) Comparison of particle size (peak), polymey disperse index and zeta potential (peak) of Lipo and MP-LP.

The morphology, size distribution, and zeta potential of liposomes and MP-LP were presented in Figure 3. Lipo and MP-LP both showed spheroidal shape with nanosize (Figure 3(A,B)). The size distribution of blank liposomes (147.7 ± 1.08 nm) and the MP-LP (157.3 ± 1.31 nm) tested by DLS indicated that the chimeric of membrane proteins increased the size of liposomes slightly (Figure 3(C)). MP-LP showed increased PDI (0.215 ± 0.009) and decreased absolute value of zeta potential (−48.3 ± 1.1) compared to the blank liposomes (PDI: 0.162 ± 0.005; zeta potential: −51.1 ± 0.6; Figure 3(C)), indicating that the membrane proteins chimeric modification may alter the dispersity and zeta potential of the nanoparticle. While it is likely for the charge of the protein, there needs future exploration to determine key proteins that mediated the alteration of zeta potentials. Nanoparticles with high zeta potentials are stable. Chimeric of membrane proteins into liposomes may affect the integrity. But the influence is minimal as the difference is marginal. More importantly, MP-LP showed good stability during storage similar to Lipo (Supplementary Table S1). In addition, coomassie blue staining showed the most membrane proteins on MP-LP (Supplementary Figure S2).

The size of MP-LP was slightly larger than free liposomes, and the protein particles on the surface of liposomes could be observed from the TEM images; also, zeta potentials of MP-LP were decreased than liposomes. Those combined results demonstrated that membrane proteins of Huh7 cells were successfully modified onto the surface of liposomes during the extruding process and formed MP-LP. A recent study also used cell membrane proteins for modification of liposomes and enhanced the tumor-targeting effects (Li et al., 2021).

### TPL loading and release

The drug-loading capacity and entrapment efficiency of TPL@MP-LP were 5.62 ± 0.80% and 68.23 ± 4.97%, respectively. For drug release, a burst release of nearly 50% of TPL was observed for both TPL@liposomes and TPL@MP-LP in the first 24 h, followed by sustained release thereafter (Supplementary Figure S3), suggesting that the liposomes and MP-LP were stable under condition (37 °C, pH 7.4) similar to circulation environment. The drug-loading capacity and entrapment efficiency are important for the translational research and industrial development of nanoliposomal formulation. Despite improved solubility of TPL after nanoliposomal loading, the amount of drugs in liposomes are not very high, and a quiet amount of free drugs were not loaded into liposomes. In our study, TPL was added into lipids for passive entrapment during liposome development. It has been reported that the drug-loading capacity can be improved via active drug encapsulation methods (Gubernator, 2011). However, as we used membrane proteins for liposome modification, we therefore applied passive loading for membrane protein protection and simplicity.

### Cellular uptake and cell viability

**Figure 4.**
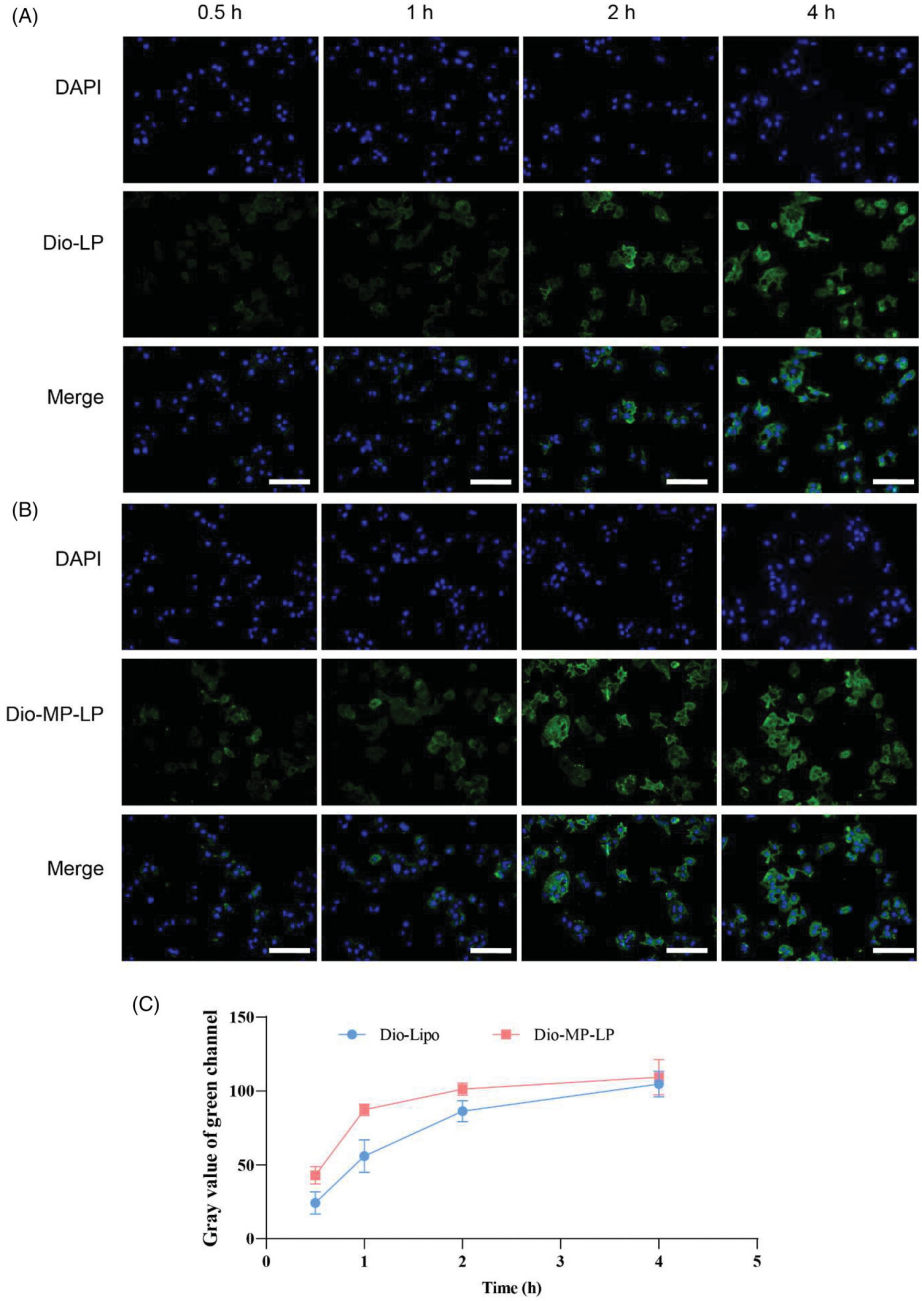
Cellular uptake study. (A) Cellular uptake of liposomes by Huh7 cells for different incubation hours. (B) Cellular uptake of MP-LP by Huh7 cells for different incubation hours. (C) Semi-quantitative analysis of cellular uptake of liposomes and MP-LP.

To compare the cellular uptake of liposomes and MP-LP, Dio-labeled liposomes and MP-LP were added at different time points to Huh7 cells for incubation. As a result, efficient and time-dependent cellular uptake was observed for both liposomes (Figure 4(A)) and MP-LP (Figure 4(B)). However, the efficiency of cellular uptake of MP-LP by Huh7 cells was significantly higher than liposomes, as observed by the semi-quantitative analysis (Figure 4(C)). MP-LP could be efficiently uptake in an hour, but it takes 2 h or more for liposomes, indicating that autologous cell membrane modification could facilitate the internalization of nanoparticles into cell. A similar phenomenon of enhanced homologous cellular uptake was also reported in other studies (Li et al., 2020), despite unclear mechanisms.

The cell viability was evaluated by CCK-8 assay. IC50 for free TPL, TPL@Lipo, and TPL@MP-LP were 15.55, 14.74, and 12.41 ng/mL, showing decreased IC50 for nanoparticles-mediated TPL delivery (Supplementary Figure S4), despite no significant statistical significance.

### Biodistribution and tumor targeting

**Figure 5.**
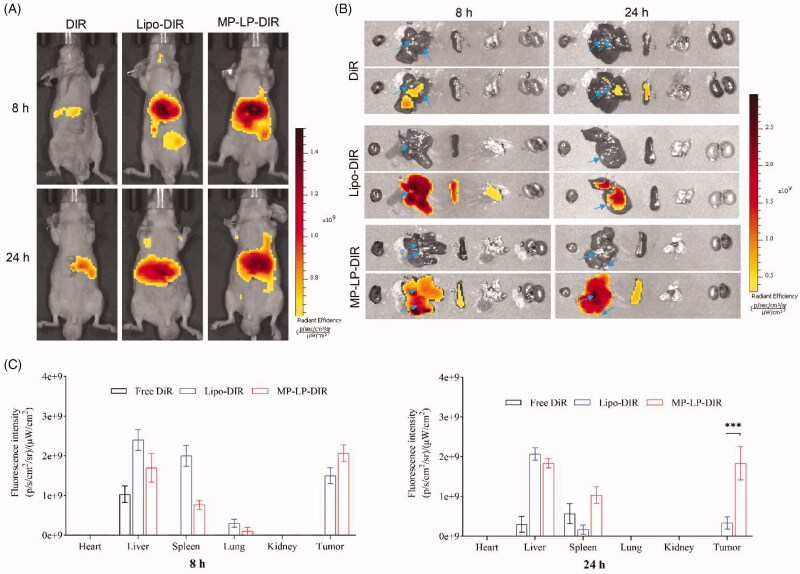
Biodistribution and tumor-targeting of nanoparticles. (A) *In vivo* biodistribution of DiR-labeled Lipo and MP-LP. (B) *Ex vivo* biodistribution and tumor accumulation of DiR-labeled Lipo and MP-LP. Blue arrow indicates the tumor site (C) Analysis of fluorescence intensity of DiR-labeled Lipo and MP-LP in major organs of HCC-bearing mice at different time points. ∗∗∗*p* < .001.

The *in vivo* biodistribution of Lipo and MP-LP was determined using a live imaging system. Tumor-bearing mice were administrated with DiR-labeled liposomes or MP-LP through tail vein injection and were imaged at 8 and 24 h post-administration (Figure 5(A)). Tumors and major organs were excised after live mice imaging. Tumor sites were observed *ex vivo* before imaging and then highlighted by arrow (Figure 5(B)). DiR-labeled Lipo accumulated mostly in liver and spleen at 8 h and can only be detected in the liver at 24 h, with minimal fluorescence intensity at the tumor site (Figure 5(B)). DiR-labeled MP-LP were observed mostly in liver and spleen similar to liposomes, but the fluorescence intensity of DiR-labeled MP-LP was significantly higher than liposomes, demonstrating enhanced tumor-targeting effects of MP-LP for membrane proteins chimeric modification (Figure 5(C)). In contrast, free DiR showed no fluorescence signals at the tumor site.

The tumor-targeting effects are of importance for the delivery of TPL for tumor therapy as TPL is highly toxic with significant side effects. While strong fluorescence signals of Lipo were observed in the liver, but Lipo showed less accumulation at the tumor site than MP-LP, demonstrating that the membrane protein modification enhanced the tumor-targeting effects of the nanoparticle. The autologous targeting or homing properties has been reported (Li et al., 2019; Villa et al., 2021). It might be difficult to specify the key protein or other components that mediate the targeting. Surface components such as membrane proteins might be important for homing properties and drug delivery (Saari et al., 2015). However, it should be noted that multiple mechanisms may be involved in the homing process.

### Antitumor efficacy of TPL@MP-LP

**Figure 6.**
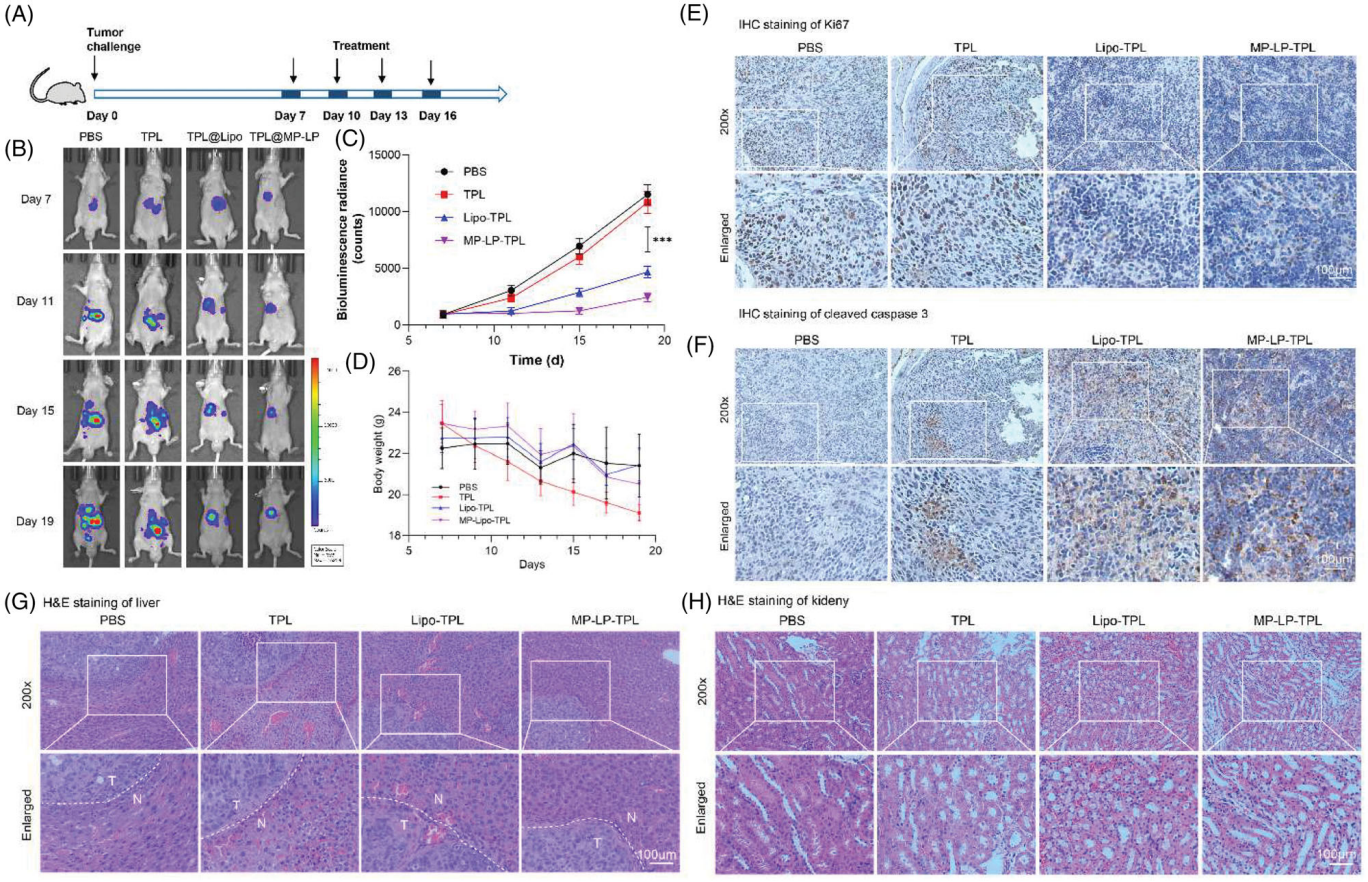
*In vivo* antitumor efficacy of TPL@MP-LP. (A) Treatment schedule of TPL@MP-LP for HCC-bearing mice. (B) Bioluminescence of HCC growth in mice (*n* = 6). (C) Quantitative analysis of bioluminescence showing tumor size in mice (*n* = 6). ∗∗∗ *p* < .001. (D) Body weight of mice before and after treatment (*n* = 6). (E) IHC analysis of Ki67 expression of the tumor site in the liver after treatments. TPL led to slightly less staining in liver tumor, and TPL@Lipo and TPL@MP-LP led to much less staining of Ki67 in liver tumor. (F) IHC analysis of cleaved caspase 3 expression of the tumor site in the liver after treatment. TPL@Lipo and TPL@MP-LP markedly improved caspase 3 expression in liver tumor. (G-H) H&E staining of liver tissues showed that TPL led to obvious liver and kidney injury, while it was not observed in the groups of Lipo-TPL and MP-LP-TPL. The bar indicated 100 μm.

For the investigation of the *in vivo* anticancer therapeutic efficacy of TPL@MP-LP on orthotopic HCC xenograft mice, tumor-bearing mice were treated with different formulations intravenously four times with an interval of 3 days (Figure 6(A)). Free TPL suppressed tumor growth slightly compared to mice receiving PBS as controls with no statistical significance. TPL-loaded liposomes exhibited enhanced antitumor efficacy, and TPL@MP-LP showed further improved therapeutic efficacy at suppressing tumor growth than free TPL (Figure 6(B,C)). The bodyweight of HCC-bearing mice was decreasing, free TPL treatment accelerated the decrease of mice body weight, demonstrating potential toxicity of TPL, but TPL@Lipo and TPL@MP-LP showed similar body weight compared to PBS controls (Figure 6(D)). Also, the IHC analysis of Ki67 (Figure 6(E)) and cleaved caspase 3 (Figure 6(F)) of the tumor site in the liver showed significantly suppressed growth and clear cell death at the tumor site for TPL@MP-LP treatment. While TPL@Lipo also showed enhance antitumor potentials, the efficacy was less significant than TPL@MP-LP treatment; this may attribute to the lower targeting efficacy of passive targeting of blank liposomes.

### Safety evaluation

Serum levels of alanine aminotransferase (ALT) and aspartate aminotransferase (AST) were analyzed to reflect liver functions; blood urea nitrogen (BUN) and creatinine (Cr) were analyzed to reflect renal functions. Tumor-bearing mice treated with TPL showed elevated levels of ALT, AST and Cr compared to other groups, demonstrating that TPL may affect liver and renal functions (Supplementary Figure S5). TPL@Lipo and TPL@MP-LP exhibited alleviated damage to the liver and renal functions, demonstrating that those nanoformulations could decrease the toxicity of TPL effectively. However, there is no difference in the toxicity between TPL@Lipo and TPL@MP-LP, demonstrating that the nanoformulation is the key to reduce the toxicity of TPL and the targeting influence more the efficacy than the toxicity. The H&E staining of major organs showed damages of free TPL to the liver and kidney, despite low TPL dose (100 μg/kg). But no significant difference among PBS, TPL@Lipo, and TPL@MP-LP groups was observed (Supplementary Figure S6).

Safety has been a major concern for therapeutic usage of TPL. During the observation, the bodyweight of HCC-bearing mice was decreasing, demonstrating that orthotopic HCC may affect the health of mice. A more remarkable decrease of body weight was noted for free TPL treatment (Figure 6(D)), demonstrating that free TPL, lacks tumor-targeting effects, caused hepatotoxicity, and nephrotoxicity as evidence by the serum analysis (Supplementary Figure S5) and HE staining of tissues (Figure 6(G,H)). However, the treatment of nanoformulations of TPL was more tolerable with less damage to other normal tissues (Supplementary Figure S6).

## Conclusions

In this study, TPL was successfully loaded into MP-LP. TPL can inhibit HCC cells proliferation and lead to apoptosis via the mitochondrial pathway, and can induce necroptosis via RIPK1/RIPK3/MLKL signaling. In particular, TPL@MP-LP showed improved cellular uptake and tumor-targeting ability. Injection of TPL@MP-LP significantly suppressed tumor growth in mice with orthotopic HCC. Also, the MP-LP-mediated delivery of TPL showed tolerable biocompatibility with low toxicity compared to that of free TPL. We demonstrated that MP-LP is a safe and efficient delivery vehicle for improving the antitumor efficacy of TPL for HCC therapy. This delivery strategy may have implications for improving outcomes of drugs highly potent but also reducing the risk of toxicity.

## Supplementary Material

Supplemental Material

## References

[CIT0001] Anwanwan D, Singh SK, Singh S, et al. (2020). Challenges in liver cancer and possible treatment approaches. Biochim Biophys Acta Rev Cancer 1873:188314. 31682895 10.1016/j.bbcan.2019.188314 PMC6981221

[CIT0002] Cartwright T, Perkins ND, L Wilson C. (2016). NFKB1: a suppressor of inflammation, ageing and cancer. Febs J 283:1812–22. 26663363 10.1111/febs.13627

[CIT0003] Crommelin DJA, van Hoogevest P, Storm G. (2020). The role of liposomes in clinical nanomedicine development. What now? Now what? J Control Release 318:256–63. 31846618 10.1016/j.jconrel.2019.12.023

[CIT0004] Daher S, Massarwa M, Benson AA, Khoury T. (2018). Current and future treatment of hepatocellular carcinoma: an updated comprehensive review. J Clin Transl Hepatol 6:69–78. 29607307 10.14218/JCTH.2017.00031 PMC5863001

[CIT0005] Elnaggar MH, Abushouk AI, Hassan AHE, et al. (2021). Nanomedicine as a putative approach for active targeting of hepatocellular carcinoma. Semin Cancer Biol 69:91–9. 31421265 10.1016/j.semcancer.2019.08.016

[CIT0006] Ferlay J, Colombet M, Soerjomataram I, et al. (2019). Estimating the global cancer incidence and mortality in 2018: GLOBOCAN sources and methods. Int J Cancer 144:1941–53. 30350310 10.1002/ijc.31937

[CIT0007] Forner A, Reig M, Bruix J. (2018). Hepatocellular carcinoma. Lancet 391:1301–14. 29307467 10.1016/S0140-6736(18)30010-2

[CIT0008] Gubernator J. (2011). Active methods of drug loading into liposomes: recent strategies for stable drug entrapment and increased in vivo activity. Expert Opin Drug Deliv 8:565–80. 21492058 10.1517/17425247.2011.566552

[CIT0009] Hou W, Liu B, Xu H. (2019). Triptolide: Medicinal chemistry, chemical biology and clinical progress. Eur J Med Chem 176:378–92. 31121546 10.1016/j.ejmech.2019.05.032

[CIT0010] Hou ZY, Tong XP, Peng YB, et al. (2018). Broad targeting of triptolide to resistance and sensitization for cancer therapy. Biomed Pharmacother 104:771–80. 29807227 10.1016/j.biopha.2018.05.088

[CIT0011] Jao HY, Yu FS, Yu CS, et al. (2016). Suppression of the migration and invasion is mediated by triptolide in B16F10 mouse melanoma cells through the NF-kappaB-dependent pathway. Environ Toxicol 31:1974–84. 26420756 10.1002/tox.22198

[CIT0012] Jiang L, Gu Y, Du Y, et al. (2021). Engineering exosomes endowed with targeted delivery of triptolide for malignant melanoma therapy. ACS Appl Mater Interfaces 13:42411–28. 34464081 10.1021/acsami.1c10325

[CIT0013] Jono H, Lim JH, Chen LF, et al. (2004). NF-kappaB is essential for induction of CYLD, the negative regulator of NF-kappaB: evidence for a novel inducible autoregulatory feedback pathway. J Biol Chem 279:36171–4. 15226292 10.1074/jbc.M406638200

[CIT0014] Kooijmans SAA, Vader P, Schiffelers RM. (2017). Tumour-bound RNA-laden exosomes. Nat Biomed Eng 1:634–6. Aug 31015606 10.1038/s41551-017-0119-4

[CIT0015] Krosch TC, Sangwan V, Banerjee S, et al. (2013). Triptolide-mediated cell death in neuroblastoma occurs by both apoptosis and autophagy pathways and results in inhibition of nuclear factor-kappa B activity. Am J Surg 205:387–96. 23428154 10.1016/j.amjsurg.2013.01.008 PMC7970732

[CIT0016] Kupchan SM, Court WA, Dailey RG, Jr, et al. (1972). Triptolide and tripdiolide, novel antileukemic diterpenoid triepoxides from Tripterygium wilfordii. J Am Chem Soc 94:7194–5. 5072337 10.1021/ja00775a078

[CIT0017] Leggio L, Arrabito G, Ferrara V, et al. (2020). Mastering the tools: natural versus artificial vesicles in nanomedicine. Adv Healthc Mater 9:e2000731. 32864899 10.1002/adhm.202000731

[CIT0018] Lener T, Gimona M, Aigner L, et al. (2015). Applying extracellular vesicles based therapeutics in clinical trials - an ISEV position paper. J Extracell Vesicles 4:30087. 26725829 10.3402/jev.v4.30087 PMC4698466

[CIT0019] Li H, Pan GF, Jiang ZZ, et al. (May 2015). Triptolide inhibits human breast cancer MCF-7 cell growth via downregulation of the ERα-mediated signaling pathway . Acta Pharmacol Sin 36:606–13. 25864647 10.1038/aps.2014.162 PMC4422943

[CIT0020] Li XJ, Jiang ZZ, Zhang LY. (2014). Triptolide: progress on research in pharmacodynamics and toxicology. J Ethnopharmacol 155:67–79. 24933225 10.1016/j.jep.2014.06.006

[CIT0021] Li Y-J, Wu J-Y, Hu X-B, et al. (2019). Autologous cancer cell-derived extracellular vesicles as drug-delivery systems: a systematic review of preclinical and clinical findings and translational implications. Nanomedicine (Lond) 14:493–509. 30694095 10.2217/nnm-2018-0286

[CIT0022] Li YJ, Wu JY, Hu XB, et al. (2019). Autologous cancer cell-derived extracellular vesicles as drug-delivery systems: a systematic review of preclinical and clinical findings and translational implications. Nanomedicine (Lond) 14:493–509. 30694095 10.2217/nnm-2018-0286

[CIT0023] Li YJ, Wu JY, Hu XB, et al. (2021). Biomimetic liposome with surface-bound elastase for enhanced tumor penetration and chemo-immumotherapy. Adv Healthc Mater e2100794. doi: 10.1002/adhm.202100794. 34160137

[CIT0024] Li YJ, Wu JY, Wang JM, et al. (2020). Gemcitabine loaded autologous exosomes for effective and safe chemotherapy of pancreatic cancer. Acta Biomater 101:519–30. 31629893 10.1016/j.actbio.2019.10.022

[CIT0025] Lim JH, Jono H, Koga T, et al. (2007). Tumor suppressor CYLD acts as a negative regulator for non-typeable Haemophilus influenza-induced inflammation in the middle ear and lung of mice. PLoS One 2:e1032. 17925880 10.1371/journal.pone.0001032 PMC2001183

[CIT0026] MacKenzie TN, Mujumdar N, Banerjee S, et al. (2013). Triptolide induces the expression of miR-142-3p: a negative regulator of heat shock protein 70 and pancreatic cancer cell proliferation. Mol Cancer Ther 12:1266–75. 23635652 10.1158/1535-7163.MCT-12-1231 PMC3707985

[CIT0027] Noel P, Von Hoff DD, Saluja AK, et al. (2019). Triptolide and its derivatives as cancer therapies. Trends Pharmacol Sci 40:327–41. 30975442 10.1016/j.tips.2019.03.002

[CIT0028] Phillips PA, Dudeja V, McCarroll JA, et al. (2007). Triptolide induces pancreatic cancer cell death via inhibition of heat shock protein 70. Cancer Res 67:9407–16. 17909050 10.1158/0008-5472.CAN-07-1077

[CIT0029] Rao S, Cronin SJF, Sigl V, Penninger JM. (2018). RANKL and RANK: from mammalian physiology to cancer treatment. Trends Cell Biol 28:213–23. 29241686 10.1016/j.tcb.2017.11.001

[CIT0030] Ren Q, Li M, Deng Y, et al. (2021). Triptolide delivery: nanotechnology-based carrier systems to enhance efficacy and limit toxicity. Pharmacol Res 165:105377. 33484817 10.1016/j.phrs.2020.105377

[CIT0031] Saari H, Lázaro-Ibáñez E, Viitala T, et al. (2015). Microvesicle- and exosome-mediated drug delivery enhances the cytotoxicity of Paclitaxel in autologous prostate cancer cells. J Control Release 220:727–37. 26390807 10.1016/j.jconrel.2015.09.031

[CIT0032] Shao J, Zaro J, Shen Y. (2020). Advances in exosome-based drug delivery and tumor targeting: from tissue distribution to intracellular fate. Int J Nanomed 15:9355–71. 10.2147/IJN.S281890 PMC7700079 33262592

[CIT0033] Siegel RL, Miller KD, Fuchs HE, Jemal A. (2021). Cancer Statistics, 2021. CA A Cancer J Clin 71:7–33. 10.3322/caac.21654 33433946

[CIT0034] Su Z, Yang Z, Xu Y, et al. (2015). Apoptosis, autophagy, necroptosis, and cancer metastasis. Mol Cancer 14:48. 25743109 10.1186/s12943-015-0321-5 PMC4343053

[CIT0035] Sun SC. (2010). CYLD: a tumor suppressor deubiquitinase regulating NF-kappaB activation and diverse biological processes. Cell Death Differ 17:25–34. 19373246 10.1038/cdd.2009.43 PMC5848464

[CIT0036] Villa A, Garofalo M, Crescenti D, et al. (2021). Transplantation of autologous extracellular vesicles for cancer-specific targeting. Research Paper. Theranostics 11:2034–47. 10.7150/thno.51344 PMC7797692 33500707

[CIT0037] Wang Y, Wang B, Yang X. (2020). The study of cellular mechanism of triptolide in the treatment of cancer, bone loss and cardiovascular disease and triptolide's toxicity. Curr Stem Cell Res Ther 15:18–23. 30834841 10.2174/1574888X14666190301155810

[CIT0038] Wu H, Wang MD, Liang L, et al. (2021). Nanotechnology for hepatocellular carcinoma: from surveillance, diagnosis to management. Small 17:e2005236. 33448111 10.1002/smll.202005236

[CIT0039] Xu L, Qiu Y, Xu H, et al. (2013). Acute and subacute toxicity studies on triptolide and triptolide-loaded polymeric micelles following intravenous administration in rodents. Food Chem Toxicol 57:371–9. 23583804 10.1016/j.fct.2013.03.044

[CIT0040] Yu D, Liu Y, Zhou Y, et al. (2020). Triptolide suppresses IDH1-mutated malignancy via Nrf2-driven glutathione metabolism. Proc Natl Acad Sci USA 117:9964–72. 32312817 10.1073/pnas.1913633117 PMC7211987

[CIT0041] Yurkin ST, Wang Z. (2017). Cell membrane-derived nanoparticles: emerging clinical opportunities for targeted drug delivery. Nanomedicine 12:2007–19. 28745122 10.2217/nnm-2017-0100 PMC5551523

[CIT0042] Zhang KL, Wang YJ, Sun J, et al. (2019). Artificial chimeric exosomes for anti-phagocytosis and targeted cancer therapy. Chem Sci 10:1555–61. 30809374 10.1039/c8sc03224f PMC6357862

