# Supplementary material for: Membrane protein-chimeric liposome-mediated delivery of triptolide for targeted hepatocellular carcinoma therapy
Source: Drug Deliv. 2021 Sep 27;28(1):2033–43. doi: 10.1080/10717544.2021.1983072 (PMC8477919; doi:10.1080/10717544.2021.1983072)
Supplement: Supplemental Material [file IDRD_A_1983072_SM3652.docx]

**Supplementary materials**

**Membrane protein-chimeric liposome-mediated delivery of triptolide for targeted hepatocellular carcinoma therapy**

Yanwen Zheng^1^, Fanfa Kong^2^, Songyang Liu^3^, Xi Liu^4^, Dongni Pei^1^, Xiongying Miao^1,*^

1. Department of Liver Surgery, The Second Xiangya Hospital of Central South University, Changsha, China

2. Institute of Hepatobiliary Diseases of Wuhan University, Transplant Centre of Wuhan University, Zhongnan Hospital of Wuhan University, Wuhan University, Wuhan, Hubei, China.

3. College of Biology, State Key Laboratory of Chemo/Biosensing and Chemometrics, Key Laboratory for Bio-Nanotechnology and Molecular Engineering of Hunan Province, Hunan University, Changsha 410082, China.

4. Department of Gastrointestinal Surgery, The Third Xiangya Hospital of Central South University, Changsha, China.

* Correspondence:

Xiongying Miao, M.D.

Department of Liver Surgery, The Second Xiangya Hospital of Central South University, Changsha 410011, China

Email: miaoxiongying3016@csu.edu.cn


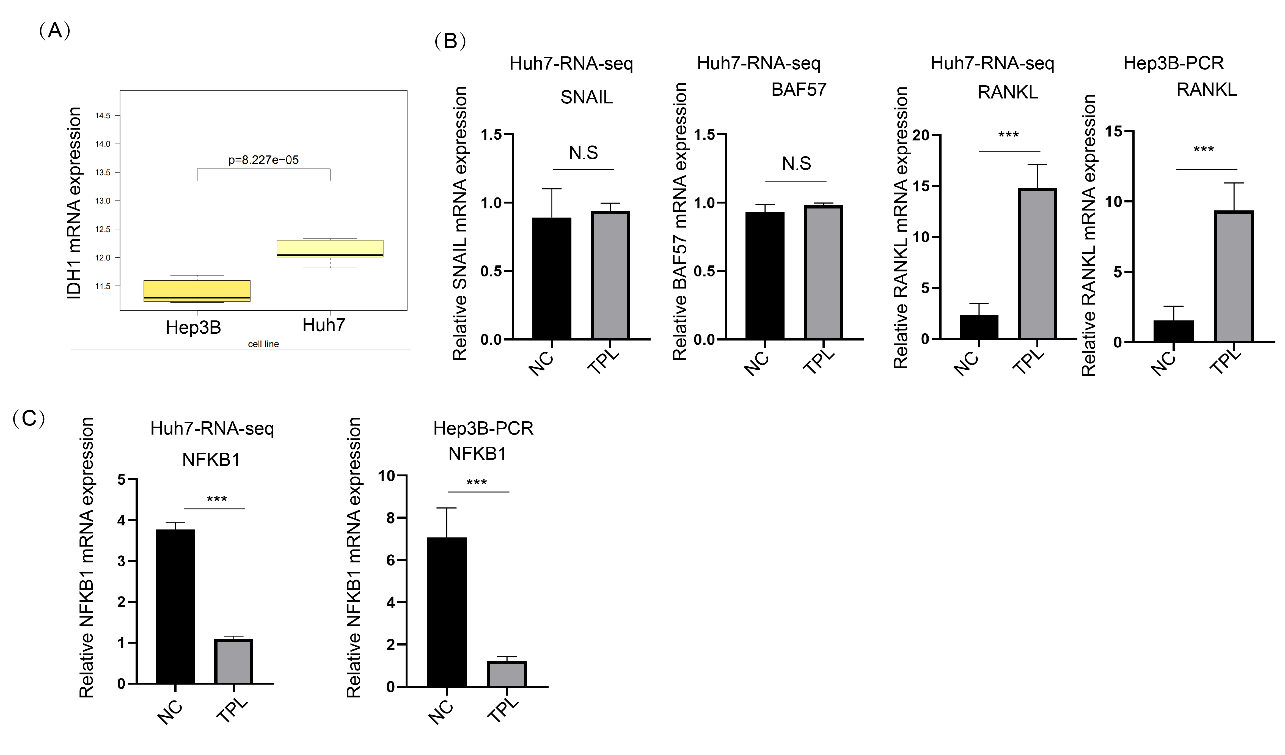


Figure S1. The difference between hep3B and Huh7 responding to TPL is regulated by expression of IDH1 and evidence for the transcriptional target for TPL on HCC cells. (A) Retrieving data from GSE151412, and comparing IDH1 mRNA between Huh7 and Hep3B cells. (B) Retrieving data from our RNA-seq and PCR, and showing that no significant difference of SNAIL and BAF57 mRNA was observed upon TPL, but RANKL was significantly increased upon TPL in HCC cells. (C) Retrieving data from our RNA-seq and PCR and showing NFKB1 mRNA was significantly decreased upon TPL in HCC cells.


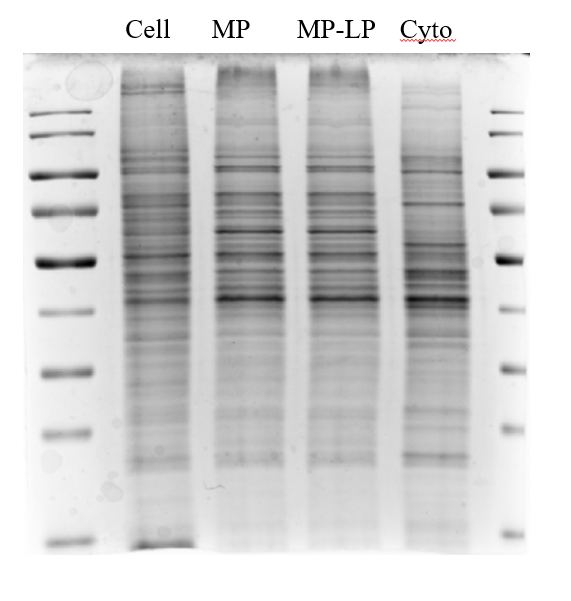
**Figure S2.** Coomassie staining analysis showing protein profiles of MP-LP. MP, membrane protein; Cyto, cytosolic proteins.


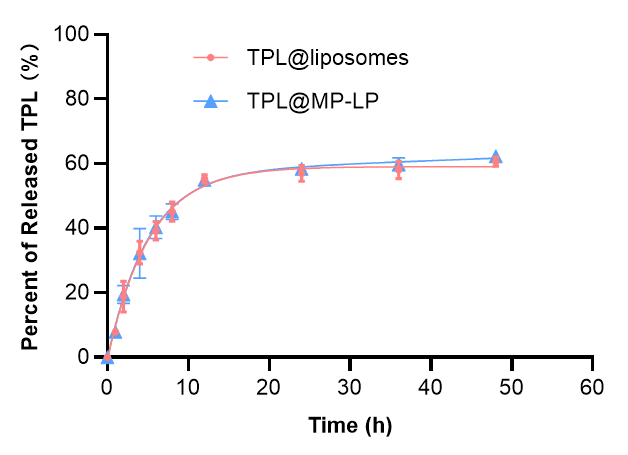


**Figure S3.** The release profile of TPL from liposomes or MP-LP.


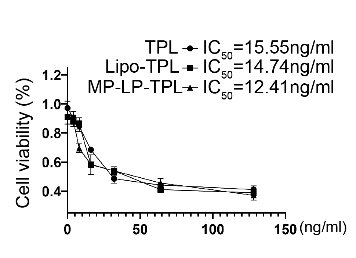


**Figure S4.** Viability of Huh7 cells tested by CCK-8 assay after 24 h of incubation with different TPL formulations. IC50 was calculated to compare the cytotoxicity.


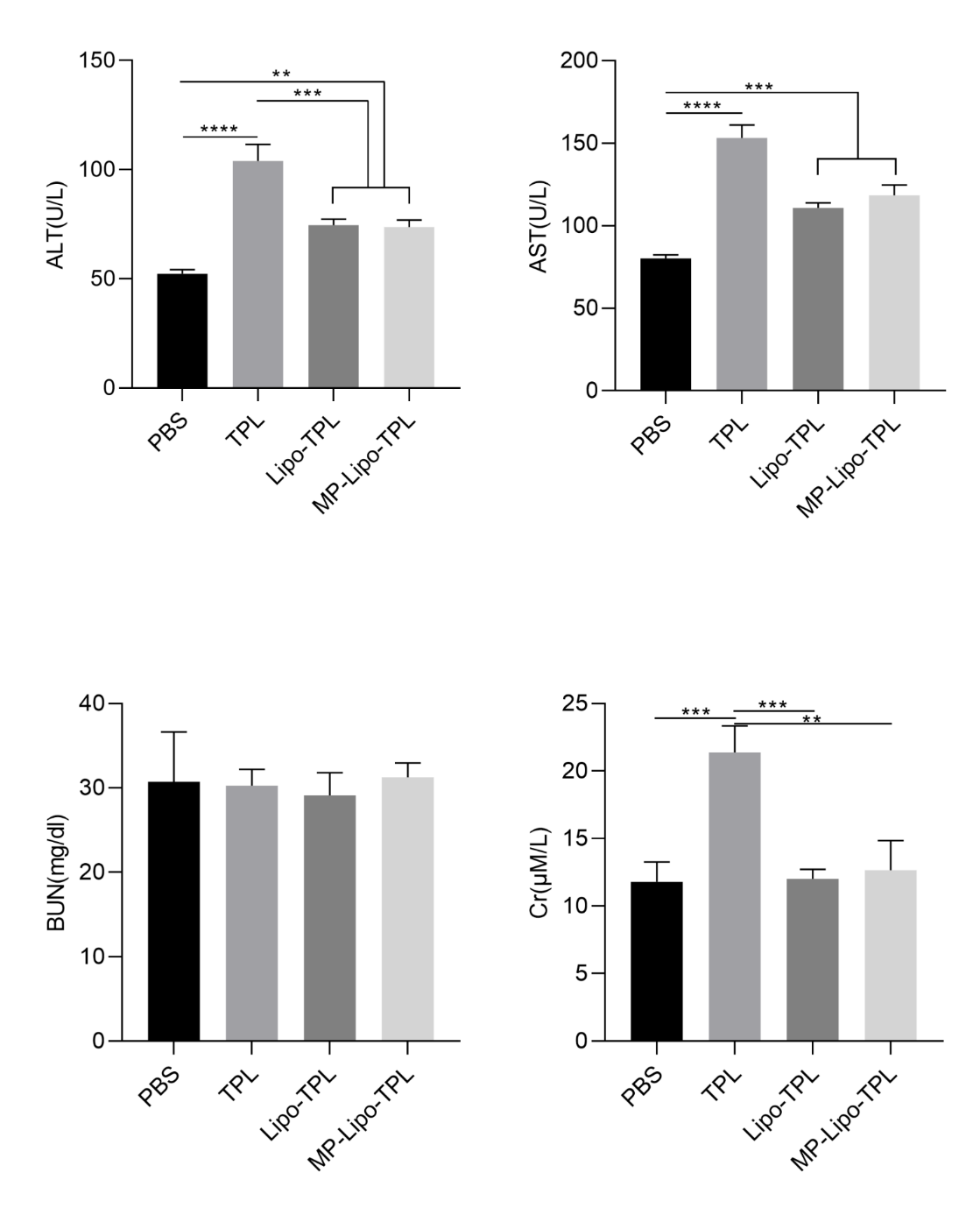


**Figure S5.** Serum levels of ALT, AST, BUN and Cr in HCC-bearing mice after treatment.


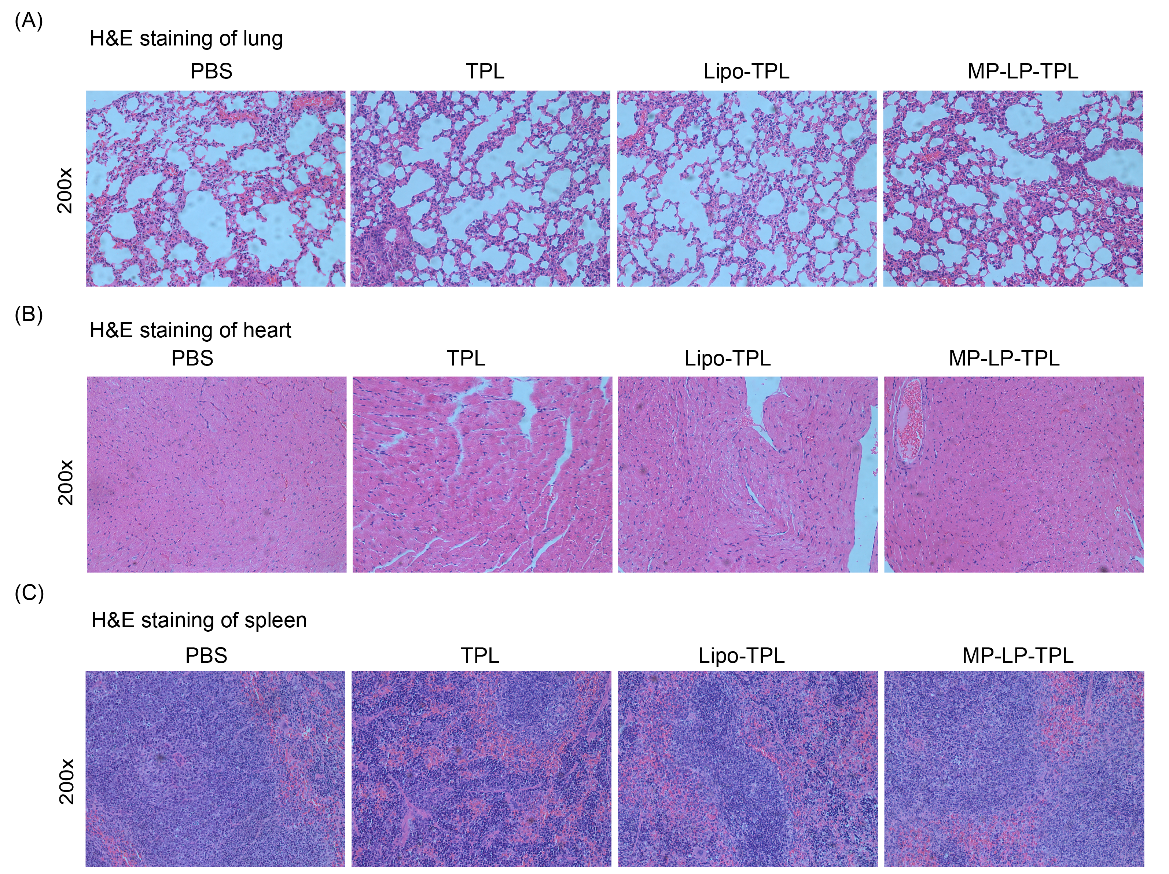


**Figure S6.** H&E staining of major organs after treatment. (A-C) No obvious side effects were observed in lung, heart and spleen induced by TPL, Lipo-TPL or MP-LP-TPL. The bar indicated 100 μm.

**Table S1.** Stability test of liposomes and MP-LP. Liposomes were stored at 4℃, MP-LP were freeze-dried and stored at -80℃.

| **Sample** | 3 days | 5 days | 7 days | 14 days | 28 days |
| --- | --- | --- | --- | --- | --- |
| Liposome | 145±0.54 nm  PDI:0.153 | 147±1.2 nm  PDI:0.143 | 146 ±0.85 nm  PDI:0.161 | 148±1.6 nm  PDI:0.174 | 150±1.24 nm  PDI:0.169 |
| MP-LP | 156±1.21 nm  PDI：0.241 | 155±0.84 nm  PDI：0.158 | 158±1.35 nm  PDI：0.254 | 159±1.25 nm  PDI：0.112 | 158±0.756  PDI：0.137 |
